# P-1918. Reduction in Antibiotic Use Due to Universal Decolonization in Nursing Homes (Secondary Analysis of the Protect Trial)

**DOI:** 10.1093/ofid/ofaf695.2087

**Published:** 2026-01-11

**Authors:** Seong Eun Kim, Gabrielle Gussin, Ken Kleinman, Thomas T Tjoa, Raveena Signh, Raheeb Saavedra, Loren G Miller, Susan Huang

**Affiliations:** Chonnam National University Medical School, GwangJu, Kwangju-jikhalsi, Republic of Korea; University of California, Irvine School of Medicine, Division of Infectious Diseases, Irvine, California; University of Massachusetts Amherst, Amherst, Massachusetts; University of California, Irvine School of Medicine, Division of Infectious Diseases, Irvine, California; University of California Irvine, Irvine, California; University of California, Irvine School of Medicine, Division of Infectious Diseases, Irvine, California; Lundquist Institute at Harbor-UCLA Medical Center, Los Angeles, CA; University of California, Irvine School of Medicine, Irvine, California

## Abstract

**Background:**

The Protect trial (NEJM 2023 PMID: 37815935) was a cluster randomized trial of 28 nursing homes that found that universal decolonization— CHG bathing and nasal iodophor— reduced infection-related hospitalizations per 1000 resident days by 31%. We evaluated whether this intervention also reduced antibiotic use.

**Methods:**

We conducted a secondary analysis of the Protect trial to evaluate antibiotics given to residents in nursing homes assigned to routine care versus decolonization. Outcome was defined as the number of days of antibiotic administration during the 7 days reported for each Minimum Data Set (MDS) assessment (admission, quarterly, discharge). We conducted a difference-in-differences logistic regression analysis using generalized linear mixed models to account for clustering within facility using an interaction term between study arm and time period.

**Results:**

Comparing the intervention to baseline period, decolonization nursing homes had a 57.5% lower odds of antibiotic prescription (OR=0.425, 95% CI: 0.403-0.448) compared to a 32.4% lower odds in routine care nursing homes (OR=0.676, 95% CI: 0.648-0.705), resulting in a 39.0% greater relative reduction (relative OR=0.610, 95% CI 0.585-0.635; p< 0.0001) after adjusting for age, gender, race, ethnicity, insurance, and coexisting conditions. Unadjusted analyses showed similar results, supporting the robustness of the findings. Predicted probabilities showed antibiotic use decreased from 6.3% to 2.8% in the decolonization group compared with a decline from 7.3% to 5.3% in the routine care group. The most frequently used antibiotic was levofloxacin (13.2% in baseline, 10.5% in intervention), followed by ceftriaxone (8.8% in baseline, 9.2% in intervention). Antibiotic use patterns are summarized in Table 1.

**Conclusion:**

These findings support universal decolonization as an effective strategy to reduce antibiotic use in nursing homes. In addition to improved health outcomes, this reduction in antibiotic use plus the previously shown reduction in infection-related hospitalizations supports universal decolonization as a cost saving strategy for nursing homes.

**Disclosures:**

Loren G. Miller, MD MPH, Armata: Grant/Research Support|GSK: Grant/Research Support|Merck: Grant/Research Support|Paratek: Grant/Research Support Susan Huang, MD, MPH, Xttrium: Conducting studies in which participating nursing homes and hospitalized patients receive contributed antiseptic products|Xttrium Laboratories: Conducting studies in which participating nursing homes and hospitalized patients receive contributed antiseptic product

